# Venous thromboembolism prophylaxis in patients with traumatic brain injury: a systematic review

**DOI:** 10.12688/f1000research.2-132.v1

**Published:** 2013-05-29

**Authors:** Yohalakshmi Chelladurai, Kent A Stevens, Elliott R Haut, Daniel J Brotman, Ritu Sharma, Kenneth M Shermock, Sosena Kebede, Sonal Singh, Jodi B Segal

**Affiliations:** 1 John Hopkins University Bloomberg School of Public Health, Baltimore, MD, 21205, USA; 2 Department of Surgery, Johns Hopkins University School of Medicine, Baltimore, MD, 21287, USA; 3 Department of Medicine, Johns Hopkins University School of Medicine, Baltimore, MD, 21287, USA; 4 Department of Pharmacy, Johns Hopkins University School of Medicine, Baltimore, MD, 21287, USA

## Abstract

**Objective: **There is considerable practice variation and clinical uncertainty about the choice of prophylaxis for preventing venous thromboembolism in patients with traumatic brain injury. We performed a systematic review to assess both the effectiveness and safety of pharmacologic and mechanical prophylaxis, and the optimal time to initiate pharmacologic prophylaxis in hospitalized patients with traumatic brain injury.

**Data sources and study selection: **MEDLINE®, EMBASE®, SCOPUS, CINAHL, International Pharmaceutical Abstracts, clinicaltrial.gov, and the Cochrane Library were searched in July 2012 to identify randomized controlled trials and observational studies reporting on the effectiveness or safety of venous thromboembolism prevention in traumatic brain injury patients.

**Data extraction: **Paired reviewers extracted detailed information from included articles on standardized forms and assessed the risk of bias in each article.

**Data synthesis:** Twelve studies (2 randomized controlled trials and 10 cohort studies) evaluated the effectiveness and safety of venous thromboembolism prophylaxis in patients with traumatic brain injury. Five of the included studies assessed the optimal timing of initiation of pharmacological prophylaxis. Low grade evidence supports the effectiveness of enoxaparin over control in reducing deep vein thrombosis. Low grade evidence also supports the safety of unfractionated heparin over control in reducing mortality in patients with traumatic brain injury. Evidence was insufficient for remaining comparisons and outcomes including the optimal timing of initiation of pharmacoprophylaxis.

**Conclusion:** There is some evidence that pharmacoprophylaxis improves deep vein thromboses and mortality outcomes in patients hospitalized with traumatic brain injury. Additional studies are required to strengthen this evidence base.

## Introduction

There is considerable practice variation and clinical uncertainty about the choice of a prophylaxis modality (pharmacologic and mechanical) and about the optimal pharmacologic agent, dose, timing of initiation, and duration for the prevention of venous thromboembolism (VTE) among patients with traumatic brain injury (TBI) ^1^. This population is at increased risk for VTE due to a combination of factors (i.e., the brain injury itself, other injuries, intensive care unit admission, immobilization, major surgery, etc.). This increased risk should prompt routine thromboprophylaxis in patients with TBI; however, the concern over an associated elevated risk of bleeding in patients with TBI often leads physicians to withhold pharmacological thromboprophylaxis. The American College of Chest Physician guidelines do not specifically address DVT prophylaxis in patients with traumatic brain injury ^2^. To help clarify the practice standards to prevent VTE events in the TBI population, we performed a comprehensive systemic review of the literature.

## Methods

The protocol for the review was developed and posted online following guidelines for systematic reviews ^3, 4^. Additional methodological details are available in our evidence report prepared for the Agency for Healthcare Research and Quality (AHRQ) ^5^.

### Data sources and search

The following databases were searched in July 2012 for primary studies: MEDLINE ^®^, EMBASE ^®^, SCOPUS, CINAHL, International Pharmaceutical Abstracts, clinicaltrial.gov, and the Cochrane Library. An analytic framework depicting our population of interest, interventions tested for prevention of VTE, intermediate and patient-oriented outcomes of treatment, as well as the harms of the interventions was developed ^3^.

### Study selection

Titles were reviewed followed by abstracts to identify randomized controlled trials (RCTs) or observational studies with comparison groups reporting on the effectiveness or safety of VTE prevention in TBI patients. Two investigators independently reviewed abstracts meeting our inclusion criteria; abstracts were excluded if both reviewers agreed that the article met one or more of the exclusion criteria ( Table 1).

**Table 1.** Inclusion/Exclusion criteria.

	Inclusion	Exclusion
**Populations**	• Human subjects (only) • Patients with traumatic brain injury	• Animal studies/models • Children • Pediatric • Adolescent • Adults in the following patient populations: • Treatment of VTE • Secondary prophylaxis • Catheter thrombosis • Antiphospholipid antibodies/other autoimmune diseases • Cancer (malignancy, chemotherapy, radiotherapy) • Cardiovascular (coronary artery bypass graft surgery, percutaneous transluminal coronary angioplasty) patients on full-dose anticoagulation • Pregnancy • Disseminated intravascular coagulation • Heparin-induced thrombocytopenia • Congenital platelet disorders • VTE prophylaxis for long distance travel • Abdominal surgery • Vascular surgery • Urological surgery • Gynecological surgery • Trauma with brain injury • Burns • Liver disease • Antiplatelet therapy • Bariatric surgery • Obese and underweight • Acute kidney injury, moderate renal impairment • Severe renal impairment, renal replacement therapy
**Intervention**	Studies that evaluate pharmacological interventions or mechanical devices	Studies of agents that have not been approved for thromboprophylaxis in the United States or interventions not available in the United States will not be evaluated
**Outcomes**	• Symptomatic deep vein thrombosis • Symptomatic pulmonary embolism • Mortality • Post-thrombotic syndrome • Quality of life • Length of hospital stay • Length of ICU stay • Bleeding (major, minor) • Allergic reaction • Mechanical device complications • Infections	No data on relevant outcomes of interest
**Type of** **study**	• Randomized controlled trials • Prospective cohort studies • Retrospective cohort studies • Case-control studies • Uncontrolled case-series for devices • Case reports of device complications • Case reports of pharmacologic therapies other than the known complications of bleeding and heparin-induced thrombocytopenia	• Case reports of efficacy • Case reports of bleeding or heparin-induced thrombocytopenia associated with pharmacologic strategies • *In vitro* studies • Animal studies • Cost-effectiveness studies • Modeling studies • Risk assessment studies • Registries without descriptions of interventions • Diagnostic studies • Ecologic study designs • Time-series designs • No original data, commentary, or editorial • Systematic reviews and meta-analysis

### Data abstraction and quality assessment

Evidence Partners 2010 web-based database management program, DistillerSR, was used to manage the screening and review process. Standardized forms for data extraction from the articles were created. Paired investigators reviewed all extracted data.

The risk of bias was assessed independently and in duplicate, using the Downs and Black instrument ^6^. Ten items that were most relevant to this review were prioritized in our assessment of risk of bias. Studies were assessed to have a low risk of bias if all of the following were true: the article completely described the hypothesis, the outcomes (in the introduction or methods section), the characteristics of the included subjects, the distribution of the potential confounders in each group, the interventions and comparisons (if relevant) the main findings, adverse events, and characteristics of the subjects lost to follow up. Additionally, we judged studies to be at low risk of bias if they randomized subjects to the intervention and concealed the assignment until randomization was complete, and if they attempted to blind the study participants and to blind those who measured the main outcomes. By this system, non-randomized studies could only be at moderate or high risk of bias. Studies were rated as having a moderate risk of bias if one of those items was not true, even if all of the others were true, or if the reporting on the distribution of potential confounders in each group was at least partially done. If two of the elements were not true, studies were rated to have a high risk of bias.

### Data synthesis and analysis

A detailed set of evidence tables was created containing all information abstracted from eligible studies. Given the substantial statistical and clinical heterogeneity, we do not report pooled results but display the individual magnitude of effect and statistical significance for the individual studies.

### Outcomes assessed

The effectiveness of pharmacological and mechanical strategies in preventing patient-oriented outcomes such as VTE, deep vein thrombosis (DVT) and pulmonary embolism (PE), mortality and progression of intracranial hemorrhage.

### Grading the evidence and applicability

The quantity, quality, and consistency of the best available evidence was graded by adapting an evidence-grading scheme recommended in the Agency for Healthcare Research and Quality: Methods Guide for Conducting Comparative Effectiveness Reviews ^7^.

## Results

**Figure 1.**
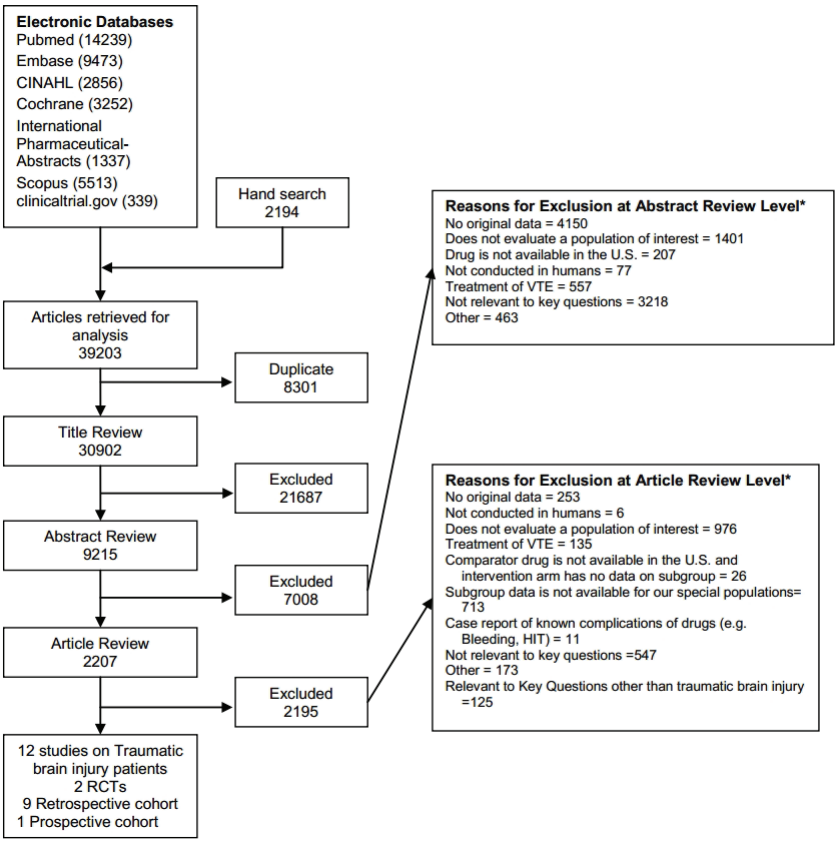
Summary of the literature search.

The literature search identified 30902 citations. After necessary exclusions and triage to other topics, 12 articles were included for this review ( Figure 1).

### Study characteristics

Seven studies that evaluated the effectiveness of pharmacological and mechanical strategies to prevent VTE in hospitalized patients with TBI were identified ^8– 14^, four that evaluated the optimal timing of initiation of pharmacological prophylaxis ^1, 15– 17^ and one study that evaluated both ^18^. Most of the studies were conducted in North America ^1, 8, 9, 11– 18^. Two RCTs were included in this review ^10, 14^. The remaining were cohort studies; nine retrospective studies ^1, 8, 9, 11, 13, 15– 18^ and one prospective ^12^. The majority of studies included patients admitted in level 1 trauma centers.

### Participant characteristics

The number of participants in the included studies ranged from 32 to 812; the mean age of the participants ranged from 36 to 47 years. The Injury Severity Score (ISS) of TBI patients was reported in eight studies; the mean ranged from 15.7 to 33.8 indicating severe multi-system trauma ^8– 12, 14, 15, 18^. The ethnicity or race of the participants was not reported in any study ( Table 2).

**Table 2.** Study characteristics.

Drug versus control										
			Patients, N		Mean Age (yrs)		Male (%)		Mean ISS Scores	
Study	Design	Comparison	Drug	Control	Drug	Control	Drug	Control	Drug	Control
*Minshall *et al.*, 2011	RETRO	Enoxaparin vs. Control	158	57	41.2	38.3	75	69	29	30.9
Salottolo *et al.*, 2010	RETRO	Enoxaparin vs. Control	255	225	48	59.5	64.3	58.5	21	16
Phelan *et al.*, 2010	RCT	Enoxaparin vs. Placebo	34	28	40.7	42.6	64	57	17.3	15.7
Kurtoglu *et al.*, 2004	RCT	Enoxaparin vs. SCD	60	60	37.1 ^¥^	37.1 ^¥^	39.2 ^¥^	39.2 ^¥^	19.5	18.3
*Minshall *et al.*, 2011	RETRO	UFH vs. Control	171	57	42	38.3	78	69	33.8	30.9
Scudday *et al.*, 2010	RETRO	UFH vs. Control	402	410	45.2	51.5	69	69	23.8	16.6
Drug versus drug										
			Patients, N		Mean Age (yrs)		Male (%)		Mean ISS Scores	
Study	Design	Comparison	Drug 1	Drug 2	Drug 1	Drug 2	Drug 1	Drug 2	Drug 1	Drug 2
Dudley *et al.*, 2010	RETRO	Dalteparin vs. Enoxaparin	159	128	45.9	47.4	72.3	77.3	35	31.3
*Minshall *et al.*, 2011	RETRO	UFH vs. Enoxaparin	171	158	42	41.2	78	75	33.8	29
SCD versus control										
			Patients, N		Mean Age (yrs)		Male (%)		Mean ISS Scores	
Study	Design	Comparison	SCD	Control	SCD	Control	SCD	Control	SCD	Control
Gersin *et al.*, 1992	PC	SCD vs. Control	14	18	38.3	36.1	71.4	77.8	30.5	32.1
Drug <72 hrs versus >72 hrs										
			Patients, N		Mean Age (yrs)		Male (%)		Mean ISS Scores	
Study	Design	Comparison	<72 h	>72 h	<72 h	>72 h	<72 h	>72 h	<72 h	>72 h
Koehler *et al.*, 2011	RETRO	Enoxaparin	268	401	39.8	40.2	69	75	27.8	29.4
Salottolo *et al.*, 2010	RETRO	Enoxaparin	108	147	NR	NR	NR	NR	NR	NR
Kim *et al.*, 2002	RETRO	UFH	47	17	37.7	44	NR	NR	30.7	35.7
Depew *et al.*, 2008	RETRO	Any heparin	29	41	NR	NR	NR	NR	NR	NR

*Study has three arms, we have shown data for all comparisons individually; UFH=Unfractionated heparin; SCD=Sequential Compression Devices; ISS=Injury Severity Score; NR=Not Reported; RCT=Randomized Controlled Trial; PC=Prospective Cohort; RETRO=Retrospective Cohort; ^¥^Mean reported for overall group.

### Intervention characteristics

Eight studies were included to assess the effectiveness and safety of pharmacological and mechanical interventions to prevent VTE in patients with traumatic brain injury ^8– 14, 18^. The interventions compared in these studies were highly heterogeneous; studies varied in drugs compared, the dosages and timing of initiation of therapy. Many studies had a control group in which active therapy was withheld from participants. The dose of pharmacological drugs used was reported in five studies; dalteparin was administered as 5000 U once daily. Unfractionated heparin (UFH) as 5000 U thrice daily, and enoxaparin as 30 mg twice daily or 40 mg daily.

Five studies independently assessed the optimal timing of the initiation of chemoprophylaxis in the same population ^1, 15– 18^. Although enoxaparin and UFH were the only pharmacological agents employed in these studies, two studies were unclear about the pharmacological agents used and were classified as “any heparin” intervention ^16, 17^. Four out of five studies compared the effectiveness and safety of pharmacoprophylaxis in preventing VTE when initiated less than 72 hours (early prophylaxis) of hospital admission versus greater than 72 hours (late prophylaxis).

There were no studies that assessed the effectiveness of inferior vena cava filters in preventing PE in TBI patients.

### Ascertainment of VTE

Most studies did not routinely screen for VTE ^1, 8– 10, 13, 14, 16, 18^. Weekly surveillance using duplex ultrasound examination was carried out in four studies ^11, 12, 15, 17^, although two of these studies performed it in high risk patients exclusively ^11, 17^.

### Outcomes

***Venous thromboembolism***. Five studies assessed the effectiveness of pharmacological agents in preventing VTE in patients with TBI ^8, 11– 13, 18^. One study demonstrated lower rates of VTE in the UFH group compared to the control group (3% vs. 1%, respectively, p=0.019) ^11^; two studies showed increased rates of VTE in the enoxaparin and sequential compression devices group compared to the control group (enoxaparin vs. control, 3.9% vs. 2.2%, p=0.29; Sequential compression devices (SCD) vs. control, 28.6% vs. 22.2%, p=0.7) ^12, 18^, while the last study demonstrated no difference in rates of VTE between dalteparin and control groups (0% vs. 0%) ^13^. Head-to-head comparison available in a study demonstrated marginally increased rates of venous thromboses in patients treated with dalteparin compared to those treated with enoxaparin (7.5% vs. 7.0%, p value not significant) ^8^.

A single study demonstrated increased rates of VTE with early enoxaparin prophylaxis when compared to late prophylaxis. (5.56% vs. 2.72% percent, Odds ratio (OR) 2.10, p=0.26) ^18^ ( Table 3).

**Table 3.** Patient-oriented outcomes.

Drug versus control											
		Patients, N		% Total DVT		% Total PE		% Total mortality		% ICH progression	
Study	Comparison	Drug	Control	Drug	Control	Drug	Control	Drug	Control	Drug	Control
*Minshall *et al.*, 2011	Enoxaparin vs. Control	158	57	1	2	0	2	5	47	5	NR
Salottolo *et al.*, 2010	Enoxaparin vs. Control	255	225	NR	NR	NR	NR	NR	NR	NR	8.4
Phelan *et al.*, 2010	Enoxaparin vs. Placebo	34	28	0	3.6	0	0	NR	NR	5.9	3.6
Kurtoglu *et al.*, 2004	Enoxaparin vs. SCD	60	60	5 ^#^	6.6 ^#^	6.6 ^¶,Φ^	3.3 ^¶,Φ^	13.3	11.6	1.6	1.6
*Minshall *et al.*, 2011	UFH vs. Control	171	57	1	2	4	2	15.8	47	12	NR
Scudday *et al.*, 2010	UFH vs. Control	402	410	NR	NR	NR	NR	0.8	3.7	3 ^¶^	6 ^¶^
Sadeh *et al.*, 2012	Dalteparin vs. Control	93	29	0	0	0	0	NR	NR	0	0
Drug versus drug											
		Patients, N		% Total DVT		% Total PE		% Total mortality		% ICH progression	
Study	Comparison	Drug 1	Drug 2	Drug 1	Drug 2	Drug 1	Drug 2	Drug 1	Drug 2	Drug 1	Drug 2
Dudley *et al.*, 2010	Dalteparin vs. Enoxaparin	159	128	NR	NR	0.6	NR	NR	NR	0	0.01
*Minshall *et al.*, 2011	UFH vs. Enoxaparin	171	158	1	1	4 ^¶^	0 ^¶^	15.8	5	12 ^¶^	5 ^¶^
SCD versus control											
		Patients, N		% Total DVT		% Total PE		% Total mortality		% ICH progression	
Study	Comparison	SCD	Control	SCD	Control	SCD	Control	SCD	Control	SCD	Control
Gersin *et al.*, 1992	SCD vs. Control	14	18	0	11.1	28.6	11.1	NR	NR	NR	NR
Drug <72 hrs versus >72 hrs											
		Patients, N		% Total DVT		% Total PE		% Total mortality		% ICH progression	
Study	Comparison	<72 h	>72 h	<72 h	>72 h	<72 h	>72 h	<72 h	>72 h	<72 h	>72 h
Koehler *et al.*, 2011	Enoxaparin	268	401	NR	NR	1.5 ^#^	2.2 ^#^	NR	NR	1.5 ^#^	1.5 ^#^
Salottolo *et al.*, 2010	Enoxaparin	108	147	NR	NR	NR	NR	NR	NR	6.5 ^#^	14.3 ^#^
Kim *et al.*, 2002	UFH	47	17	4.3 ^#^	5.9 ^#^	4.3 ^#^	0 ^#^	8.5 ^#^	5.9 ^#^	NR	NR
Depew *et al.*, 2008	Any heparin	29	41	10.4	14.6	3.5	0	NR	NR	3.5	3.8

*Study has three arms; UFH=Unfractionated heparin; SCD=Sequential Compression devices; DVT=Deep vein thrombosis; PE= Pulmonary embolism; ICH=intracranial hemorrhage; N=Number; NR=Not Reported; ^#^p value not significant; ^¶^p value significant; ^Φ^- Of the total PE, 6.6% in the enoxaparin arm and 3.3% in the IPC arm were fatal; ^Ж^- DVT risk per 100 patients.

Overall, the evidence was concluded to be insufficient to comment on the effectiveness and optimal timing of initiation of VTE prophylaxes in TBI patients ( Table 4).

**Table 4.** Strength of evidence.

Intervention	Outcome	Studies N	Enrolled participants	Risk of bias	Directness	Summary precision	Consistency	Strength of evidence and magnitude of effect
**Enoxaparin** **vs. SCD/** **control**	VTE ^18^	1	480	High	Direct	Imprecise	Unknown	Insufficient evidence to comment on effectiveness of Enoxaparin vs. SCD/control in reducing Total VTE in TBI patients. 3.9% vs. 2.2%; p=0.29
	DVT ^1, 9, 14^	3	397	Moderate	Direct	Imprecise	Consistent	***Low grade evidence to suggest that enoxaparin*** ***reduces DVT in TBI patients when compared to SCD/*** ***control***
	PE ^1, 9, 14^	3	397	Moderate	Direct	Imprecise	Inconsistent	Insufficient evidence to comment on effectiveness of enoxaparin vs. SCD/control in reducing Total PE in TBI patients
	Mortality ^1, 14^	2	182	Moderate	Direct	Imprecise	Inconsistent	Insufficient evidence to comment on effectiveness of enoxaparin vs. SCD/control in reducing Total mortality in TBI patients
	Progression of ICH ^1, 14^	2	182	Moderate	Direct	Imprecise	Inconsistent	Insufficient evidence to comment on effectiveness of enoxaparin vs. SCD/control/placebo in reducing Exacerbation of epidural hematoma in TBI patients
**UFH vs.** **control**	VTE ^11^	1	812	High	Direct	Precise	Unknown	Insufficient evidence to comment on effectiveness of UFH vs. control in reducing Total VTE in TBI patients. 1% vs. 3%; p=0.019
	DVT ^9^	1	228	High	Direct	Unknown	Unknown	Insufficient evidence to comment on effectiveness of UFH vs. control in reducing Total DVT in TBI patients. 1% vs. 2%*
	PE ^9^	1	228	High	Direct	Unknown	Unknown	Insufficient evidence to comment on effectiveness of UFH vs. control in reducing Total PE in TBI patients. 4% vs. 2%*
	Mortality ^9, 11^	2	1040	High	Direct	Precise	Consistent	***Low grade evidence to suggest that UFH reduces*** ***mortality in TBI compared to controls***
**Dalteparin vs. control**	VTE ^13^	1	122	High	Direct	Unknown	Unknown	Insufficient evidence to comment on effectiveness of dalteparin vs. control in reducing Total VTE in TBI patients. 0% vs. 0%*
	Progression of ICH ^13^	1	122	High	Direct	Unknown	Unknown	Insufficient evidence to comment on effectiveness of dalteparin vs. control in reducing progression of ICH in TBI patients. 0% vs. 0%*
**Enoxaparin** **vs. UFH**	DVT ^9^	1	329	High	Direct	Unknown	Unknown	Insufficient evidence to comment on effectiveness of enoxaparin vs. UFH in reducing Total DVT in TBI patients. 1% vs. 1%*
	PE ^9^	1	329	High	Direct	Precise	Unknown	Insufficient evidence to comment on effectiveness of enoxaparin vs. UFH in reducing Total PE in TBI patients. 0% vs. 4%; p<0.05
	Mortality ^9^	1	329	High	Direct	Precise	Unknown	Insufficient evidence to comment on effectiveness of enoxaparin vs. UFH in reducing Total mortality in TBI patients. 5% vs. 15.8%; p<0.05
	Progression of ICH ^9^	1	329	High	Direct	Precise	Unknown	Insufficient evidence to comment on effectiveness of enoxaparin vs. UFH in reducing progression of ICH in TBI patients. 5% vs. 12%; p<0.05
**Enoxaparin** **vs.** **dalteparin**	VTE ^8^	1	287	Moderate	Direct	Imprecise	Unknown	Insufficient evidence to comment on effectiveness of enoxaparin vs. dalteparin in reducing Total VTE in TBI patients. 7% vs. 7.5%; p=0.868
	Progression of ICH ^8^	1	287	Moderate	Direct	Unknown	Unknown	Insufficient evidence to comment on effectiveness of enoxaparin vs. dalteparin in reducing progression of ICH in TBI patients. 0.08% vs. 0%*
**SCD vs.** **control**	VTE ^12^	1	32	High	Direct	Imprecise	Unknown	Insufficient evidence to comment on effectiveness of SCD vs. control in reducing Total VTE in TBI patients. 28.6% vs. 22.2%; p=0.7
	PE ^12^	1	32	High	Direct	Unknown	Unknown	Insufficient evidence to comment on effectiveness of SCD vs. control in reducing Total PE in TBI patients. 28.6% vs. 11.1%*
**Enoxaparin** **<72 hrs. vs.** **>72 hrs.**	VTE ^18^	1	480	High	Direct	Imprecise	Unknown	Insufficient evidence to comment on effectiveness of enoxaparin started <72 hrs vs. >72 hrs in reducing VTE in TBI patients. 5.6% vs. 2.7%; p=0.26
	DVT ^1^	1	699	High	Direct	Imprecise	Unknown	Insufficient evidence to comment on effectiveness of enoxaparin started <72 hrs vs. >72 hrs in reducing proximal DVT in TBI patients. 1.5% vs. 3.5%; p=0.12
	PE ^1^	1	669	High	Direct	Imprecise	Unknown	Insufficient evidence to comment on effectiveness of enoxaparin started <72 hrs vs. >72 hrs in reducing PE in TBI patients. 1.5% vs. 2.2%; p=0.49
	Progression of ICH ^1, 18^	2	924	High	Direct	Imprecise	Inconsistent	Insufficient evidence to comment on effectiveness of enoxaparin started <72 hrs vs. >72 hrs in reducing progression of ICH in TBI patients
**UFH <72** **hrs. vs. >72** **hrs.**	DVT ^15^	1	64	High	Direct	Imprecise	Unknown	Insufficient evidence to comment on effectiveness of UFH started <72 hrs vs. >72 hrs in reducing DVT in TBI patients. 4.3% vs. 5.9%; p=1.00
	PE ^15^	1	64	High	Direct	Imprecise	Unknown	Insufficient evidence to comment on effectiveness of UFH started <72 hrs vs. >72 hrs in reducing PE in TBI patients. 4.3% vs. 0%; p=0.96
	Mortality ^15^	1	64	High	Direct	Imprecise	Unknown	Insufficient evidence to comment on effectiveness of UFH started <72 hrs vs. >72 hrs in reducing total mortality in TBI patients. 8.5% vs. 5.9%; p=1.00

UFH=Unfractionated heparin; SCD=Sequential Compression devices; NR=Not Reported; NS=Not significant; ^*^P-values or tests of statistical significance not reported; # Two sided P-estimated using Fishers exact test. Bold-italic text indicates studies with evidence for effectiveness. Non bold-italic text indicates studies with insufficient evidence.

***Deep vein thrombosis***. Four studies were included to evaluate the efficacy of enoxaparin, UFH and sequential compression devices in preventing the development of DVT in patients with TBI ^9, 10, 12, 14^. A single study demonstrated reduced rates of DVT in enoxaparin and UFH heparin groups compared to control (1% vs. 1% vs. 2% respectively, p value not reported) ^9^. Two more studies demonstrated lower rates of DVT in patients treated with enoxaparin compared to those treated with placebo and sequential compression devices (0% vs. 3.6%, p=0.45 and 5% vs. 6.6%, p=0.07) ^10, 14^. In contrast to this, a fourth study demonstrated that patients treated with sequential compression devices experienced fewer events when compared to a control group (0% vs. 11.1%) ^12^.

In two “any heparin” studies, the rates of DVT were consistently higher in the late prophylaxis group ^16, 17^. The same was observed in patients treated with UFH; rates of DVT were higher when UFH was commenced later than 72 hours (4.3% vs. 5.9%, p value not significant) ^15^ ( Table 3).

Three individual studies demonstrated that rates of DVT were lower in patients treated with enoxaparin when compared to controls or patients treated with sequential compression devices only ^9, 10, 14^. Consistent, direct, yet imprecise results, which included one RCT with a low risk of bias, led to the conclusion that low-grade evidence supported the effectiveness of enoxaparin over control/sequential compression devices in reducing DVT in hospitalized patients with TBI. However, the evidence is insufficient to comment on the optimal timing of initiation of chemoprophylaxis in the same population ( Table 4).

***Pulmonary embolism***. Five out of the eight included studies assessed the effectiveness of prophylaxis with enoxaparin, dalteparin, UFH and sequential compression devices in preventing development of PE in patients hospitalized with TBI. The results of these studies were equivocal. One study demonstrated that patients treated with enoxaparin failed to develop PE, whilst those in the control and UFH intervention groups did, the rate being lower in the control group ^9^. In contrast, a RCT demonstrated that there was no difference in rates of PE in enoxaparin-treated patients and controls (0% vs. 0%) ^14^. Two studies showed varying outcomes in patients treated with sequential compression devices only; a RCT demonstrated lower rates of PE, all of which were fatal, in this group compared to treatment with enoxaparin (3.3% vs. 6.6%, p=0.04) ^10^. However, in another study, the patients in the sequential compression devices intervention group were reported to have experienced an increase in pulmonary embolic events in comparison to control patients (28.6% vs. 11.1%, p value not reported) ^12^. The last study reported the rate of development of PE in patients treated with dalteparin only, limiting an assessment of comparative effectiveness ^8^.

Optimal timing of initiation of chemoprophylaxis in TBI populations to prevent development of PE was analyzed in three studies. Two studies demonstrated increased incidence of PE with early prophylaxis (3.5% vs. 0% and 4.3% vs. 0%), whereas in the third study, patients treated with enoxaparin within 72 hours of admission experienced fewer pulmonary embolic events (1.5% vs. 2.2%, respectively, p=0.49) ^1, 17, 18^ ( Table 3).

The evidence was concluded to be insufficient to comment on the effectiveness and optimal timing of initiation of prophylaxes in preventing PE in TBI patients ( Table 4).

***Total mortality***. Three studies included in this review evaluated the efficacy of prophylaxis with UFH or enoxaparin versus no prophylaxis or treatment with sequential compression devices only. Two studies uniformly demonstrated increased mortality in control groups when compared to patients treated with enoxaparin and UFH ^9, 10^. However, the third study demonstrated that rates of mortality were increased in patients treated with enoxaparin when compared to those prescribed sequential compression devices only (13.3% vs. 11.6%, p=0.08) ^10^.

A single cohort study reported increased deaths with early UFH prophylaxis when compared to late prophylaxis (8.5% vs. 5.9%, p=1.0) ^15^ ( Table 3).

Low grade evidence supported the effectiveness of UFH over no pharmacoprophylaxis in reducing total mortality in patients hospitalized with traumatic brain injury ( Table 4).

***Progression of intracranial hemorrhage***. The rates of progression of intracranial hemorrhage resulting from prophylaxis with dalteparin, enoxaparin, or UFH were reported in six studies ^8– 11, 13, 14^. Two studies reported that there was no difference in rates of progression of intracranial hemorrhage between the control or sequential compression devices only group and the pharmacoprophylaxis (enoxaparin and dalteparin) group ^10, 13^. Another set of two studies that compared prophylaxis with UFH and enoxaparin to control or placebo demonstrated equivocal results ^11, 14^; patients treated with UFH had lower rates of progression of intracranial hemorrhage, while those treated with enoxaparin had higher rates. Two other studies demonstrated head-to-head comparisons of two pharmacological agents. According to one study, patients treated with enoxaparin and dalteparin had comparable rates of intracranial bleeding (0.001% vs. 0%) ^8^, while the other demonstrated a statistically significant increase in intracranial bleed in patients treated with UFH compared to those treated with enoxaparin (12% vs. 5%, p<0.05) ^9^.

Three studies evaluating the optimal timing of initiation of pharmacoprophylaxis reported on rates of progression of intracranial hemorrhage in TBI populations ^1, 15, 18^. Even though all three studies reported increased rates of intracranial hemorrhage when prophylaxis was initiated with enoxaparin or any other heparin after 72 hours of admission, the increase was only minimal in two studies (3.5% vs. 3.8%; 1.46% vs. 1.54%) ( Table 3).

Overall, the evidence was insufficient to comment on the effect of pharmacological and mechanical prophylaxis and timing of initiation of pharmacoprophylaxis on progression of intracranial bleeding in TBI patients ( Table 4).

### Risk of bias

Of the twelve studies included in this review, only one RCT was at a low risk of bias ^14^. With the exception of a single cohort study that was at a moderate risk of bias ^8^, ten were estimated to be high risk of bias studies. Most cohort studies had incomplete descriptions of the important confounders and a lack of adjustment for differences between groups. They also had incomplete accounts of losses to follow-up. All of these are important confounders and threaten the internal validity of these studies.

### Applicability

The participants that these studies recruited were typical of participants admitted to other trauma centers and hence findings are generalizable. The studies were generally representative of patients with TBI in the USA. Gender was inconsistently reported, thus we could not assess the applicability of these findings to females. We did not have details to assess the applicability of this evidence to other racial groups since the studies inconsistently reported on ethnicity or race. Some studies excluded patients with previous VTE ^1, 10^ as well as those at higher risk of bleeding, such as those with low platelet counts ^1, 10, 14, 15^, limiting generalizability to these high-risk subgroups.

## Discussion

We found low-grade evidence that enoxaparin reduced rates of DVT and UFH reduced rates of mortality when compared to no pharmacoprophylaxis in TBI patients. The evidence was insufficient to comment on the effectiveness and safety of remaining comparators. Evidence was also insufficient for assessment of optimal timing of initiation of pharmacoprophylaxis for all comparators and outcomes.

We found only two RCTs that addressed VTE prophylaxis in patients with TBI. The remaining studies were single-center cohort studies, the majority of which were retrospective, having high risk of bias. Although the studies in this review asked similar questions (i.e., enoxaparin vs. heparin, pharmacologic prophylaxis vs. SCDs) and had similar patient populations, the scarcity of good quality studies with low risk of biases prevents definitive conclusions.

We identified a retrospective cohort study by Kwiatt *et al.* with a moderate risk of bias, published after our search cutoff date that evaluated the effectiveness of enoxaparin compared to control in reducing venous thrombosis and progression of intracranial hemorrhage in TBI patients ^19^. The results of this study were consistent with other studies included in our review that compared enoxaparin with a control or placebo group. This study demonstrated that the rates of venous thrombosis and progression of intracranial hemorrhage were significantly higher in patients treated with enoxaparin compared to patients in the control group (9.1% vs. 3.1% and 42% vs. 24% respectively, p<0.001 for both outcomes) indicating a potential for more harm than benefit with utilization of enoxaparin in this population. This reiterates the need for good quality studies to establish the effectiveness and safety of VTE prophylaxis in patients with TBI.

Our results should be interpreted in the context of other systematic reviews and existing guidelines. We did not identify any existing systematic reviews about the role of VTE prophylaxis and its optimal timing and initiation in patients with traumatic brain injury. The two organizations, The Eastern Association for the Surgery of Trauma (EAST) and the Brain Trauma Foundation, that provide guidelines for the care of trauma patients and patients with traumatic brain injury, respectively, do not make specific recommendations about DVT prophylaxis in TBI patients. EAST practice guidelines address DVT prophylaxis in the general trauma patient but do not make specific recommendations about patients with brain trauma. In 2007, the Brain Trauma Foundation Guidelines for the Management of Severe Traumatic Brain Injury found no good quality data to support the use of DVT prophylaxis in TBI patients. They found level III evidence for IPC and chemoprophylaxis, while stating that “ *there is insufficient evidence to support recommendations regarding the preferred agent, dose, or timing of pharmacologic prophylaxis for deep vein thrombosis (DVT)*” ^20^.

Additionally, the American College of Chest Physician guidelines do not specifically address DVT prophylaxis in these patients ^2^.

### Limitations

Our systematic review identified important weaknesses in the literature. We did not identify high quality RCTs for this review. The majority of observational studies included in this review were at a high risk of bias and did not report on several quality items of interest. The studies were heterogeneous in the definition of VTE and bleeding outcomes precluding any meaningful pooling in a meta-analysis. We also did not find data on several pharmacologic comparisons of interest or details about optimal timing of initiation of prophylaxis in this population. We were unable to assess the possibility of publication bias or selective outcomes reporting and its impact on our findings.

### Future research

Studies among patients with TBI are needed to determine whether pharmacologic DVT prophylaxis should be employed in these patients and the timing of administration. Studies should also determine the role of appropriate classification and severity of TBI when deciding to administer pharmacologic prophylaxis. Our report shows that confounding by indication was a major problem in these studies. Patients at high risk for thrombotic outcomes were more likely to receive prophylaxis and more likely to have events-the treated and untreated patients were not comparable. Future studies should consider the use of appropriate analytic strategies such as instrumental variables that control for unobserved variables if an appropriate instrument can be identified for analysis. High-quality observational studies that control for confounding by indication, such as provider and practice patterns, and confounding by disease severity may be needed as RCTs typically exclude or do not report on these populations.

## Conclusion

Low grade evidence supports the effectiveness of enoxaparin over no pharmacoprophylaxis in reducing the rates of DVT in patients with TBI. Low-grade evidence also supported the safety of UFH over no pharmacoprophylaxis in reducing total mortality in the same population. The evidence was insufficient for the remaining comparators and outcomes assessed such as VTE and PE.
