# Supplementary material for: Venous thromboembolism prophylaxis in patients with traumatic brain injury: a systematic review
Source: F1000Res. 2013 May 29;2:132. [Version 1] doi: 10.12688/f1000research.2-132.v1 (PMC4184319; doi:10.12688/f1000research.2-132.v1)
Supplement: Peer review history

# Peer review: Referee response for version 1

**DOI:** 10.5256/f1000research.1383.r4154

**Published:** 2014-04-08

**Authors:** Ewout Steyerberg, Maryse Cnossen

**Affiliations:** 1 Department of Public Health, Erasmus Medical Center, Rotterdam, Netherlands; 2 Erasmus Medical Center, Rotterdam, Netherlands

The paper is well written and the tables and figures are clear. The conclusion is well based on the results.

There is a recent review that was related to the current review. Phelan (2012) conducted a critical literature review about pharmacologic venous thromboembolism prophylaxis after traumatic brain injury. The method by Phelan was less systematic, and he only included pharmacological prophylaxis (instead of also mechanical). However, 9 of the included studies in this review were also reviewed in the study by Phelan. Overall, the current study is therefore quite similar to the review by Phelan.

The main conclusion remains that further research is urgently needed in this area.

Some specific comments:

**Introduction**

- The authors state that “this population is at increased risk for VTE due to a combination of factors (i.e. the brain injury itself, other injuries, intensive care unit admission, immobilization, major surgery etc.)”. They do not mention a source here. Perhaps a subgroup of TBI patients at risk for VTE, however, not all patients will be at risk (e.g. uncomplicated mTBI patients).
- In the introduction I miss some information about pharmacological and mechanical prophylaxis (what is it, when is it used, examples etc).

 

**Methods**

- No patient and injury characteristics are mentioned as inclusion criteria, however, GCS may be an important confounding factor in the research question. The search terms and mesh terms are not mentioned. It is not mentioned whether papers were excluded  if published before a certain date.
- In table 1 authors mention a long list of population exclusion criteria. This seems in contrast with the ISS score > 15 in all studies, indicating multi-system trauma. Some more information is necessary here (how did the authors handle studies in which some of the patients met the exclusion criteria?).
- It is not clear to me what is meant by “trauma with brain injury”.
- Also, underweight and obesity are mentioned as exclusion criteria. How did the authors account for this? BMI is not often reported in studies examining TBI.

 

**Results**

- The injury severity score is used to indicate severity. However, this score does not account for severity of TBI. Do the studies report GCS scores?
- In the table with study characteristics, the study by Sadeh is not included it seems?

 

**Discussion**

- An extra limitation is that the authors excluded studies that were comparing drugs not available in the US (n=26).

We have read this submission. We believe that we have an appropriate level of expertise to confirm that it is of an acceptable scientific standard.

# Peer review: Referee response for version 1

**DOI:** 10.5256/f1000research.1383.r1353

**Published:** 2013-08-07

**Authors:** David Bar-Or

**Affiliations:** 1 Ampio Pharmaceuticals, Greenwood Vlg, CO, USA

The systematic review of Chelladurai *et al.* on VTE prophylaxis in patients with traumatic brain injury is an excellent review and analysis of the literature on the subject. It reinforces the urgent need for well controlled, prospective studies to assess the safety, efficacy and drug choice for thromboprophylaxis in this group of patients. Timing of intervention, severity and type of injury and associated conditions, interruption of treatment for surgical procedures, effects on the geriatric population and others are variables that would require special attention. The evidence reported in this review, as the authors conclude, supports (although weak) the use of thromboprophylaxis in this group of patients. It is probable that a subgroup may benefit more than others and it would be interesting to focus even a retrospective study to such a group.

I have read this submission. I believe that I have an appropriate level of expertise to confirm that it is of an acceptable scientific standard.
